# Supplementary material for: Asynchronous magnetic resonance elastography: Shear wave speed reconstruction using noise correlation of incoherent waves
Source: Magn Reson Med. 2022 Oct 27;89(3):990–1001. doi: 10.1002/mrm.29502 (PMC9792433; doi:10.1002/mrm.29502)
Supplement: Supplementary file 1 — DATA S1 MATLAB code used to generate the numerical simulations of Figure 1 [file MRM-89-990-s002.zip › k-Wave/helpfiles/makeBall.html]

makeBall :: Functions (k-Wave)


# makeBall

Create a binary map of a filled ball within a 3D grid.

## Syntax

```
ball = makeBall(Nx, Ny, Nz, cx, cy, cz, radius)
ball = makeBall(Nx, Ny, Nz, cx, cy, cz, radius, plot_ball)
```

## Description

`makeBall` creates a binary map of a filled ball within a three-dimensional grid (the ball position is denoted by 1's in the matrix with 0's elsewhere). A single grid point is taken as the disc centre thus the total diameter of the ball will always be an odd number of grid points. The plot that is displayed after calling `makeBall(64, 64, 64, 32, 32, 32, 20, true);` is given below.

## Inputs

|  |  |
| --- | --- |
| `Nx, Ny, Nz` | size of the 3D grid [grid points] |
| `cx, cy, cz` | centre of the ball [grid points] |
| `radius` | ball radius [grid points] |

## Optional Inputs

|  |  |
| --- | --- |
| `plot_ball` | Boolean controlling whether the ball is plotted using `voxelPlot` (default = false) |

## Outputs

|  |  |
| --- | --- |
| `ball` | 3D binary map of a filled ball |

## Examples

- Simulations In Three Dimensions
- Photoacoustic Waveforms in 1D, 2D and 3D
- 3D FFT Reconstruction For A Planar Sensor
- 3D Time Reversal For A Planar Sensor
- 3D Time Reversal For A Spherical Sensor

## See Also

`makeCircle`, `makeDisc`, `makeSphere`
